# Hierarchical Disruption of the Tryptophan–Melatonin Axis Contributes to Glioma Progression Through AKT/ERK/STAT3 Signalling

**DOI:** 10.1111/jcmm.71243

**Published:** 2026-06-12

**Authors:** Bo Tan, Suqiu Yao, Shuangyin He, Tao Chen, Han Chen, Wenfu Yang, Xiyuan Tang, Tingting Xu, Jiajie Zhang, Xiaohong Yin, Ying Chen, Peng Song

**Affiliations:** ^1^ Department of Neurosurgery Guangyuan Central Hospital Guangyuan Sichuan China; ^2^ Department of Endocrinology Guangyuan Central Hospital Guangyuan Sichuan China

**Keywords:** AKT signalling, *ASMT*, ERK pathway, glioma, melatonin, molecular mechanism, *MTNR1A*, STAT3, tumour progression

## Abstract

Melatonin signalling, mediated by membrane receptors and tightly regulated biosynthetic enzymes, is a key component of circadian and neuroendocrine control in the brain. However, whether the tryptophan–melatonin axis remains hierarchically intact during glioma progression and how its disruption affects downstream signalling remain unclear. In this study, transcriptomic data from TCGA, CGGA, and GTEx were integrated to characterize the expression patterns of melatonin receptors (MTNR1A and MTNR1B) and biosynthetic enzymes (AANAT and ASMT) across normal brain tissue, lower‐grade glioma and glioblastoma. Protein expression was validated by immunohistochemistry, and functional consequences were investigated through gain‐ and loss‐of‐function experiments in glioma cells, followed by proliferation, migration, invasion, apoptosis and signalling analyses. Multi‐layered analyses revealed a coordinated disruption of the tryptophan–melatonin axis during glioma progression. Expression of AANAT, ASMT, MTNR1A and MTNR1B progressively declined with increasing tumour grade and was associated with poor prognosis. Immunohistochemistry confirmed reduced MTNR1A and ASMT protein expression in glioma tissues. Restoration of these factors suppressed glioma cell proliferation, migration and invasion while promoting apoptosis. Mechanistically, these effects were accompanied by inhibition of AKT, ERK and STAT3 signalling. These findings demonstrate that hierarchical disruption of receptor‐ and synthesis‐dependent melatonin signalling is a defining molecular feature of glioma and may contribute to malignant progression through activation of AKT/ERK/STAT3 pathways, providing new insights into the biological and therapeutic relevance of the tryptophan–melatonin axis in glioma.

## 1 Introduction

Melatonin is a multifunctional indoleamine that plays a central role in circadian regulation, neuroendocrine signalling and cellular homeostasis within the central nervous system [1, 2]. Beyond its systemic secretion by the pineal gland, melatonin is also synthesized locally in the brain, where it exerts both receptor‐dependent and receptor‐independent actions [1, 3]. Classical melatonin signalling is mediated by the G protein–coupled receptors MTNR1A (MT1) and MTNR1B (MT2), which regulate intracellular cyclic AMP, MAPK/ERK, and PI3K/AKT signalling cascades [4, 5]. In parallel, endogenous melatonin availability is governed by a tightly regulated biosynthetic pathway, in which arylalkylamine N‐acetyltransferase (AANAT) and acetylserotonin O‐methyltransferase (ASMT) catalyse the conversion of serotonin to melatonin [1, 6]. This dual‐layer organization—receptor signalling capacity and synthetic competence—forms the structural basis of melatonin signalling integrity in the human brain.

Tryptophan metabolism represents a critical metabolic decision point in neural tissues, balancing flux between serotonin–melatonin synthesis and alternative catabolic routes such as the kynurenine pathway [7]. Perturbations of this balance have broad biological consequences, influencing oxidative stress, immune regulation and cellular metabolism [6, 8]. Accumulating evidence suggests that disruption of melatonin signalling, whether through receptor loss or impaired synthesis, may shift tryptophan utilization towards immunosuppressive and pro‐oxidative pathways, thereby altering the neuroimmune and metabolic microenvironment [7, 9, 10]. Despite increasing interest in the role of melatonin in neurological and oncological contexts, how receptor‐dependent and synthesis‐dependent components of the melatonin axis are hierarchically altered in human brain diseases remains poorly characterized.

Gliomas represent a spectrum of primary brain tumours arising within the central nervous system and provide a unique human disease model in which circadian, metabolic and signalling pathways are profoundly dysregulated [11, 12, 13]. While previous studies have reported anti‐proliferative and cytoprotective effects of melatonin in experimental glioma models, a systematic evaluation of melatonin‐axis integrity in human glioma tissues–integrating transcriptomic architecture, protein‐level validation and downstream signalling consequences–has been lacking [14, 15, 16, 17, 18]. In this study, we used human glioma as a pathological context to investigate hierarchical remodelling of the tryptophan–melatonin axis. By integrating large‐scale transcriptomic analyses with protein validation and functional interrogation of key melatonin‐axis components, we aimed to define how loss of receptor‐ and synthesis‐dependent melatonin signalling reshapes downstream signalling pathways in the human brain.

## 2 Methods

### 2.1 Study Design and Biological Rationale

**FIGURE 1:**
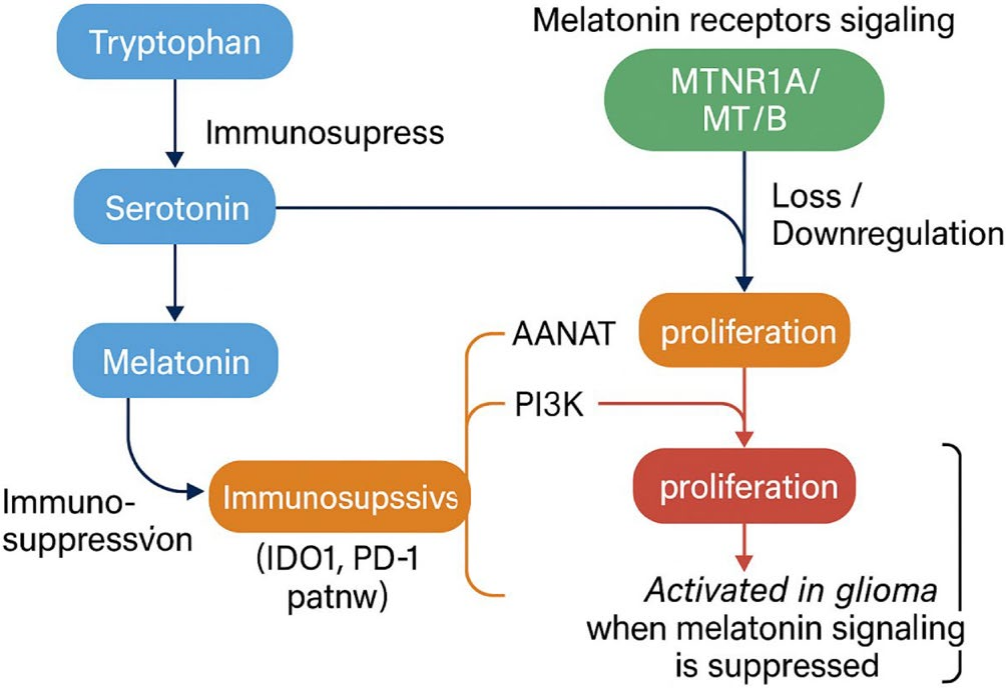
Schematic overview of tryptophan–melatonin pathway remodelling in glioma. Diagram illustrating the metabolic conversion of tryptophan to serotonin and melatonin via AANAT and ASMT, and signalling through melatonin receptors MTNR1A (MT1) and MTNR1B (MT2). The alternative kynurenine pathway mediated by IDO/TDO is also shown. In glioma, reduced expression of *MTNR1A* and *ASMT* is associated with impaired melatonin signalling and enhanced activation of oncogenic and immune‐modulatory pathways, including PI3K/AKT, ERK/MAPK and AHR‐related signalling.

This study was designed to evaluate the integrity of the tryptophan–melatonin signalling axis in the human brain under pathological conditions, using glioma as a disease model. Rather than focusing solely on tumour–normal contrasts, we aimed to systematically characterise receptor‐dependent and synthesis‐dependent components of melatonin signalling and to assess their downstream signalling consequences. An integrative strategy combining transcriptomic profiling, protein‐level validation, cell‐based functional assays and pathway analyses was applied to delineate hierarchical remodelling of the melatonin axis in human glioma. A schematic overview of the tryptophan–melatonin metabolic cascade, receptor‐mediated signalling and hypothesised downstream pathway interactions in glioma is shown in Figure 1.

### 2.2 Human Transcriptomic Datasets and Tissue Sources

Publicly available, de‐identified RNA sequencing datasets were obtained from The Cancer Genome Atlas (TCGA) glioblastoma (GBM) and lower‐grade glioma (LGG) projects, as well as from the Genotype‐Tissue Expression (GTEx) consortium, which served as a reference for normal human brain tissue. Only samples with complete expression data for melatonin‐axis genes were included. After harmonisation, the final cohort comprised 163 GBM samples, 518 LGG samples (517 for *AANAT*) and 207 normal brain samples. All datasets were accessed in compliance with their respective data use policies.

### 2.3 Selection and Quantification of Melatonin‐Axis Genes

Key components of the melatonin signalling axis were selected a priori based on established biological roles: the membrane receptors *MTNR1A* (MT1) and *MTNR1B* (MT2) and the biosynthetic enzymes arylalkylamine N‐acetyltransferase (*AANAT*) and acetylserotonin O‐methyltransferase (*ASMT*). Gene expression levels were quantified as transcripts per million (TPM). For visualization and comparative analyses, log1p‐transformed TPM values were used, while raw TPM values were retained for reporting of group medians and interquartile ranges to preserve biological interpretability.

### 2.4 Data Preprocessing and Cross‐Dataset Harmonization

TCGA and GTEx datasets were merged by intersecting gene symbols and annotated with group identifiers (normal brain, LGG, GBM). To minimize the impact of technical variability between datasets, analyses focused on non‐parametric, within‐gene comparisons rather than parametric cross‐dataset modelling. All biologically plausible zero expression values were retained. No winsorization or outlier exclusion was applied. The robustness of observed expression patterns was evaluated by comparing distributional features and effect sizes across groups.

### 2.5 Protein‐Level Validation by Immunohistochemistry

Protein expression of melatonin‐axis components, including MTNR1A, MTNR1B, AANAT and ASMT, was assessed by immunohistochemistry (IHC) in human normal brain tissue, lower‐grade glioma and glioblastoma specimens. Representative sections were stained using validated primary antibodies under standardized conditions. The primary antibodies used for IHC were as follows: anti‐MTNR1A antibody, Shanghai Huzhen Biotechnology Co. Ltd., catalogue no. Hz‐R0027, dilution 1:200; anti‐MTNR1B antibody, Shanghai Enzyme‐linked Biotechnology Co. Ltd., catalogue no. ml086273, dilution 1:100; anti‐AANAT antibody, Shanghai Huzhen Biotechnology Co. Ltd., catalogue no. Hz‐R3914, dilution 1:200; and anti‐ASMT antibody, Wuhan AmyJet Scientific Inc. / Abbexa, catalogue no. abx126833, dilution 1:200. Staining intensity was evaluated semi‐quantitatively, and group‐level differences were assessed to confirm whether transcriptomic alterations were reflected at the protein level.

### 2.6 Cell‐Based Functional Assays Targeting Melatonin‐Axis Components

To investigate the biological consequences of melatonin‐axis disruption, *MTNR1A* and *ASMT* expression were experimentally manipulated in glioma cell lines using overexpression and knockdown approaches. Changes in protein expression were confirmed by immunoblotting. Cellular behaviours associated with melatonin signalling were evaluated using CCK‐8 proliferation assays, wound‐healing migration assays, Transwell invasion assays, and flow cytometry–based apoptosis analyses. These assays were selected to assess functional responses linked to melatonin‐mediated cellular regulation rather than nonspecific cytotoxicity.

### 2.7 Pathway and Signalling Analyses

To explore downstream signalling consequences of melatonin‐axis remodelling, gene set variation analysis (GSVA) and gene set enrichment analysis (GSEA) were performed using curated KEGG and Hallmark gene sets. Pathway activity scores were correlated with melatonin‐axis gene expression to identify signalling pathways associated with receptor‐ and synthesis‐dependent melatonin signalling. Experimental validation of key pathways was conducted by western blotting and quantitative PCR (qPCR), focusing on AKT, ERK, and STAT3 signalling nodes. Phosphorylated and total protein levels were assessed to distinguish changes in pathway activation from changes in protein abundance.

### 2.8 Statistical Analysis

All statistical analyses were performed using R software version 4.4.0. Data are presented as mean ± standard deviation (SD) unless otherwise indicated. Normality was assessed using the Shapiro–Wilk test. For normally distributed data, comparisons between two groups were conducted using unpaired two‐tailed Student's *t*‐test, whereas comparisons among multiple groups were performed using one‐way analysis of variance (ANOVA) followed by Tukey's post hoc test. For non‐normally distributed transcriptomic data, pairwise comparisons were performed using the two‐sided Wilcoxon rank‐sum test or Mann–Whitney U test. Survival analysis was conducted using Kaplan–Meier analysis with the log‐rank test. Correlation analyses were performed using Pearson's or Spearman's correlation coefficient, as appropriate. For gene set enrichment analysis, pathways with a false discovery rate (FDR) < 0.05 were considered statistically significant. Multiple‐comparison correction was performed using the Benjamini–Hochberg false discovery rate method where applicable. A *p* value < 0.05 was considered statistically significant.

### 2.9 Biological Validation and Study Limitations

This study integrates transcriptomic profiling with protein‐level validation and functional interrogation to assess the integrity of melatonin signalling in a human brain disease context. However, several limitations should be acknowledged. First, circadian timing and temporal dynamics of melatonin signalling were not explicitly evaluated. Second, in vivo validation was beyond the scope of the present study. Despite these limitations, the convergent evidence from transcriptomic, protein, functional, and signalling analyses supports a coherent model of hierarchical melatonin‐axis disruption and its downstream signalling consequences.

### 2.10 Ethical Considerations and Data Availability

All bioinformatic analyses were conducted using publicly available, de‐identified datasets. Experimental validation was performed using archived anonymized human glioma tissue specimens and established glioma cell lines. No prospective human recruitment or animal experiments were involved in this study. The use of archived human specimens was approved by the institutional ethics committee, and the requirement for informed consent was waived in accordance with institutional policies and national regulations. Processed data and analysis scripts are available from the corresponding author upon reasonable request.

## 3 Results

### 3.1 Transcriptomic Remodelling of the Tryptophan–Melatonin Axis Across Human Brain and Glioma

To define the integrity of the melatonin signalling axis in human brain disease, we first analysed the transcriptomic expression of melatonin receptors (*MTNR1A, MTNR1B*) and biosynthetic enzymes (*AANAT, ASMT*) across normal brain tissue, lower‐grade glioma (LGG) and glioblastoma (GBM) using integrated TCGA, CGGA, and GTEx datasets. Expression levels were quantified as transcripts per million (TPM) and compared across cohorts using non‐parametric statistical approaches to mitigate batch‐related effects, with cohort‐ and gene‐specific sample sizes summarised in Table 1.

**TABLE 1:** Sample sizes for melatonin‐axis transcript analysis across cohorts.

Group	GBM Tumour (T)	LGG Tumour (T)	Normal brain (*n*)
*n* (*ASMT/MTNR1A/MTNR1B*)	163	518	207
*n* (*AANAT*)	163	517	207

*Note:* Sample sizes (n) for expression analyses of *MTNR1A, MTNR1B, ASMT*, and *AANAT* in glioblastoma (GBM) tumours, lower‐grade glioma (LGG) tumours and normal brain tissues are shown. Minor differences in sample numbers between genes reflect dataset‐specific missing values in RNA sequencing profiles.

**FIGURE 2:**
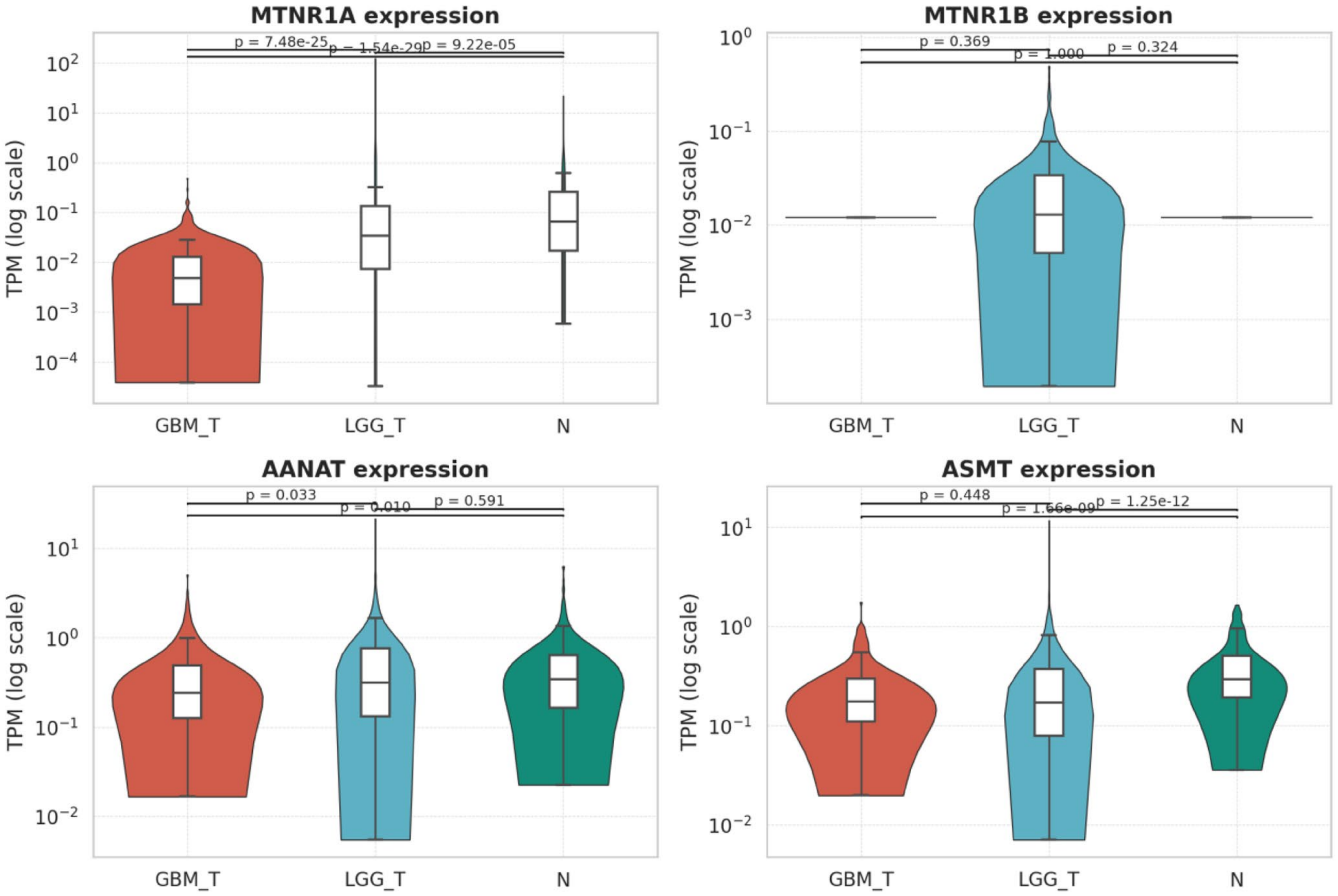
Transcriptomic distributions of melatonin‐axis genes across normal brain and glioma. Raincloud‐style violin–box plots with jittered observations illustrate the distribution of transcript per million (TPM) values for *MTNR1A, MTNR1B, AANAT*, and *ASMT* in normal brain tissue (N), lower‐grade glioma (LGG‐T) and glioblastoma (GBM‐T). Expression values are shown on a log1p scale. Boxes indicate the interquartile range (IQR) with median lines, and individual points represent samples. Pairwise comparisons among groups were performed using two‐sided Wilcoxon rank‐sum tests, with Benjamini–Hochberg false discovery rate correction where applicable. Adjusted *p* values are annotated in the figure.

**FIGURE 3:**
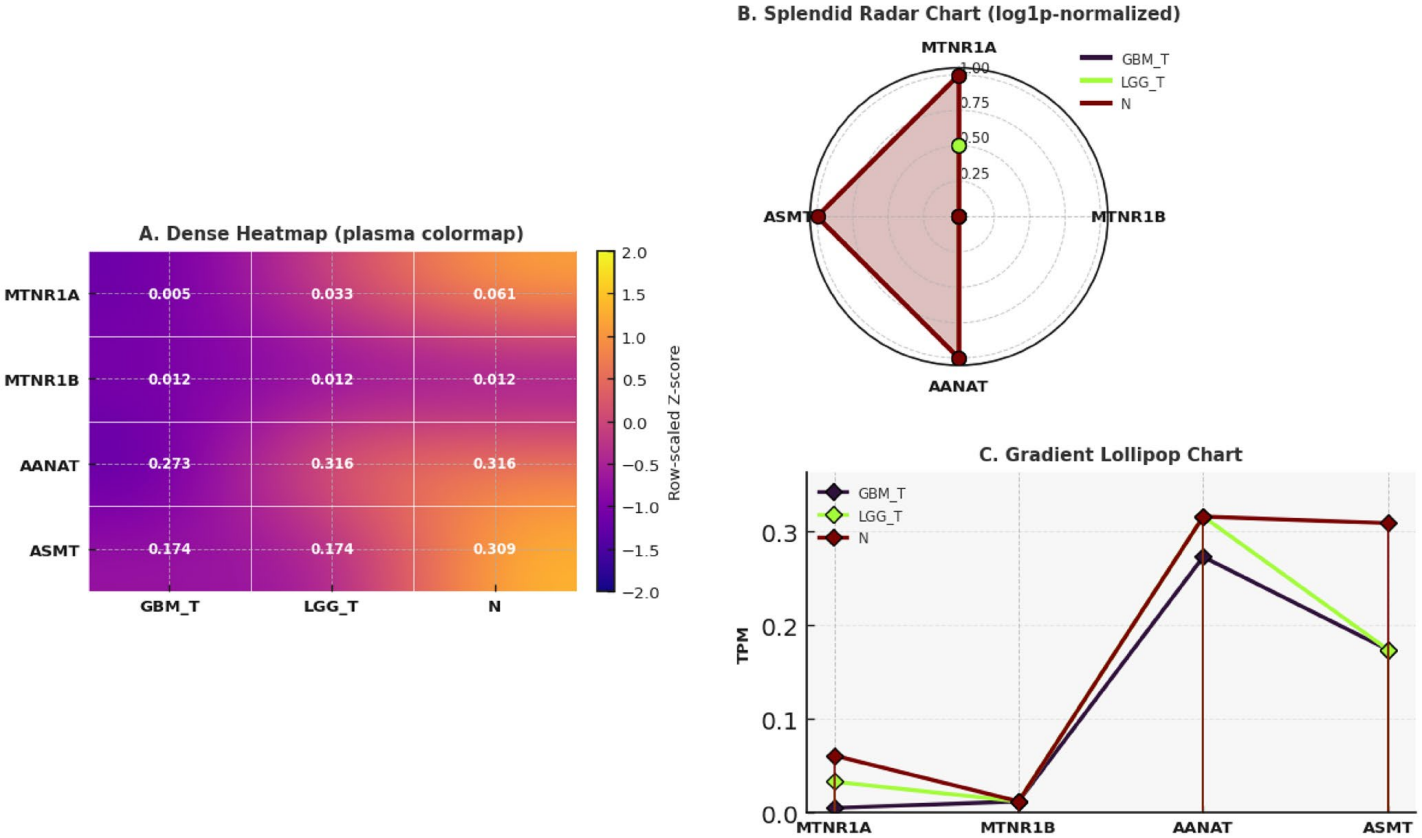
Composite visualization of melatonin‐axis expression across glioma groups. Left panel: Heatmap of row‐scaled median TPM values for melatonin‐axis genes, with raw median expression values overlaid. Top right: Radar plots depicting log1p‐normalized median expression profiles across normal brain (N), lower‐grade glioma (LGG‐T) and glioblastoma (GBM‐T). Bottom right: Lollipop‐style plots comparing group‐wise median TPM values for each gene. Together, these visualizations highlight relative and absolute expression differences among melatonin receptors and biosynthetic enzymes. This figure provides descriptive visualization of group‐wise median expression values; no additional statistical testing was performed in this figure.

Distinct, grade‐dependent alterations in melatonin‐axis components were observed. *MTNR1A* expression showed a marked reduction in GBM compared with both LGG and normal brain tissue, with intermediate levels retained in LGG. In contrast, *MTNR1B* transcripts were uniformly low across all groups, although greater variability was noted in LGG samples. Among biosynthetic enzymes, *ASMT* exhibited significant downregulation in both LGG and GBM relative to normal brain, whereas *AANAT* expression was largely preserved in LGG and only modestly reduced in GBM. These findings indicate that glioma progression is accompanied by a hierarchical remodelling of the melatonin axis, characterized by preferential loss of receptor‐dependent signalling capacity related to MTNR1A and synthesis‐limiting enzymatic activity (*ASMT*), rather than a uniform suppression of all melatonin‐related components. Gene‐wise transcriptomic distributions and pairwise group comparisons are shown in Figure 2. Integrated visual summaries emphasizing relative and absolute expression differences across glioma grades are presented in Figure 3. Group‐wise median TPM values with interquartile ranges are provided in Table 2, and statistical significance for pairwise cohort comparisons is detailed in Table 3.

**TABLE 2:** Group‐wise median transcript per million (TPM) values and interquartile ranges for melatonin‐axis genes.

Gene	GBM T	LGG T	Normal (N)
*MTNR1A*	0.005 (0.005–0.061)	0.033 (0.005–0.089)	0.061 (0.005–0.102)
*MTNR1B*	0.012 (0.012–0.012)	0.012 (0.012–0.082)	0.012 (0.012–0.012)
*AANAT*	0.273 (0.099–0.439)	0.316 (0.099–0.606)	0.316 (0.148–0.571)
*ASMT*	0.174 (0.095–0.303)	0.174 (0.081–0.344)	0.309 (0.174–0.470)

*Note:* Expression levels of *MTNR1A, MTNR1B, AANAT*, and *ASMT* are reported as median TPM with interquartile ranges (IQR) for glioblastoma (GBM) tumours, lower‐grade glioma (LGG) tumours and normal brain tissues. TPM values are shown in their raw form for interpretability, whereas log1p‐transformed values were used for graphical visualization.

**TABLE 3:** Statistical significance of pairwise group comparisons for melatonin‐axis gene expression.

Gene	GBM T vs. N	LGG T vs. N	GBM T vs. LGG T
*MTNR1A*	~0	0.002	~0
*MTNR1B*	0.022	~0	~0
*AANAT*	0.016	0.954	0.004
*ASMT*	~0	~0	0.641

*Note:* Two‐sided Mann–Whitney U test *p* values for pairwise comparisons between glioblastoma (GBM) tumours, lower‐grade glioma (LGG) tumours and normal brain tissues are shown for each melatonin‐axis gene. Extremely small *p* values (e.g., < 1 × 10^−10^) are denoted as ‘~0’. Multiple‐comparison adjustments were performed using the Benjamini–Hochberg false discovery rate method, with all key comparisons remaining significant after correction.

### 3.2 Protein‐Level Validation Confirms Hierarchical Loss of Melatonin‐Axis Components in Glioma Tissues

To determine whether transcriptomic alterations translated into protein‐level changes, immunohistochemical (IHC) analyses were performed on human normal brain, lower‐grade glioma and glioblastoma specimens. Consistent with transcriptomic findings, MTNR1A protein expression was robust in normal brain tissue, reduced in LGG and markedly diminished in GBM. MTNR1B protein expression was generally weak across all tissue types, with minimal staining observed in high‐grade tumours.

**FIGURE 4:**
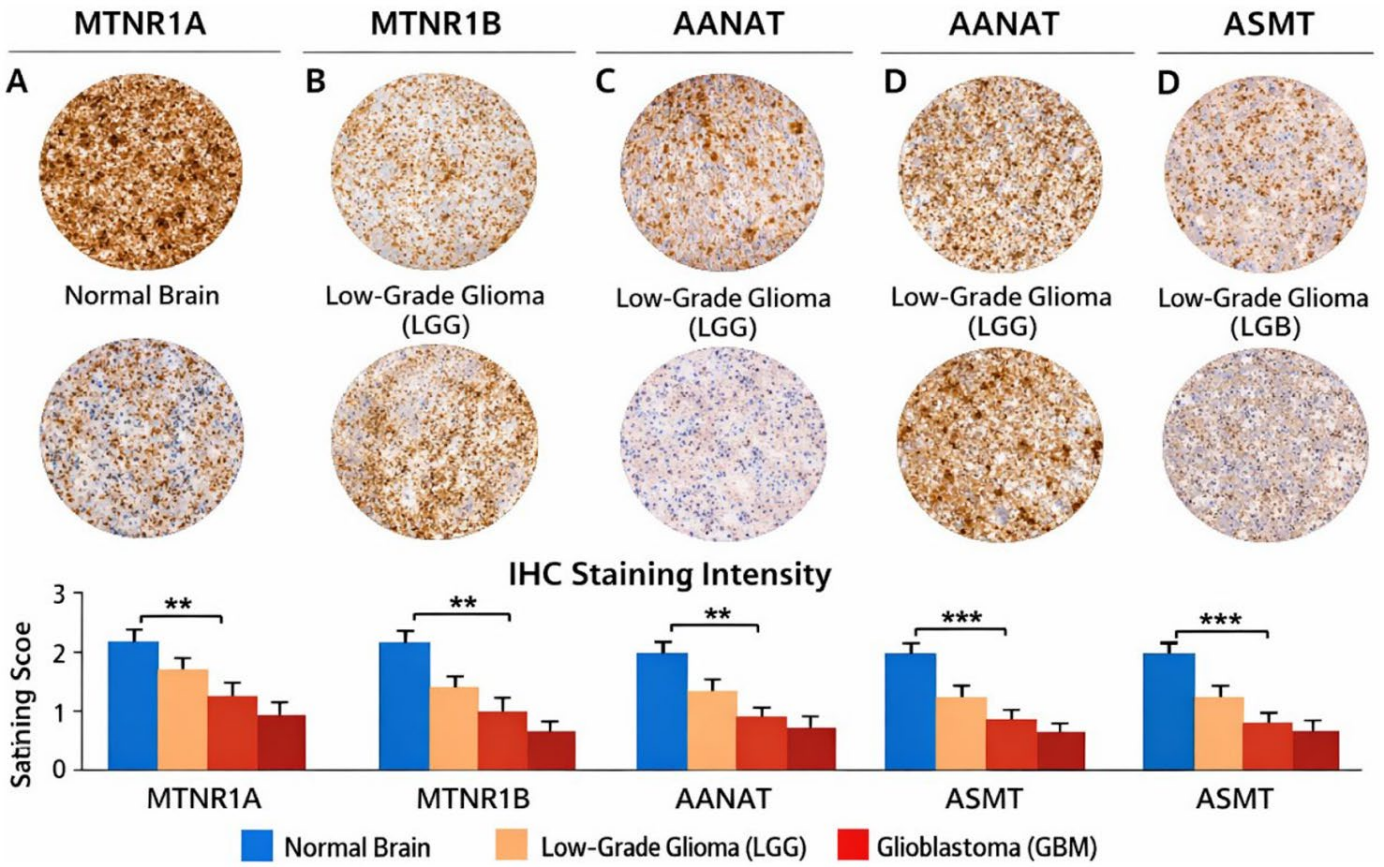
Immunohistochemical validation of melatonin‐axis components in normal brain and glioma. Panel A: Immunohistochemistry (IHC) staining of MTNR1A in normal brain and glioma tissue. Representative images of MTNR1A expression in normal brain (top), low‐grade glioma (middle) and glioblastoma (GBM) tissue (bottom). The staining intensity varies across tissue types, with notable downregulation in GBM compared to normal brain. Panel B: IHC staining of MTNR1B showing similar results, with lower expression in glioma tissues compared to normal brain, particularly in GBM. Panel C: IHC staining of AANAT demonstrating differential expression across tissue types, with significantly reduced levels in GBM compared to normal brain and low‐grade glioma. Panel D: IHC staining of ASMT showing decreased expression in both low‐grade glioma and GBM compared to normal brain, highlighting the altered melatonin axis in tumour tissues. Bar Graph: Quantification of IHC staining intensity for each marker (*MTNR1A, MTNR1B, AANAT*, and *ASMT*) across normal brain, low‐grade glioma and glioblastoma tissues. Statistical comparisons among the three groups were performed using one‐way ANOVA followed by Tukey's post hoc test. Data are presented as mean ± SD. *p < 0.05, **p < 0.01.

Analysis of melatonin biosynthetic enzymes further supported a hierarchical pattern of disruption. AANAT protein expression remained detectable in LGG specimens but was reduced in GBM, whereas ASMT protein expression was substantially decreased in both LGG and GBM compared with normal brain tissue. Quantitative scoring of IHC staining intensity confirmed statistically significant reductions in MTNR1A and ASMT protein expression with increasing tumour grade. Together, these protein‐level data validate the transcriptomic observation that receptor‐dependent and synthesis‐dependent melatonin signalling components are differentially compromised in human glioma. Representative immunohistochemical staining patterns and semi‐quantitative analyses are shown in Figure 4.

### 3.3 Functional Perturbation of *MTNR1A* and *ASMT* Alters Cellular Behaviours Associated With Melatonin Signalling

To explore the biological consequences of melatonin‐axis disruption, *MTNR1A* and *ASMT* expression were experimentally manipulated in glioma cell lines using overexpression (OE) and knockdown (KD) approaches. Successful modulation of MTNR1A and ASMT protein levels was confirmed by immunoblotting.

**FIGURE 5:**
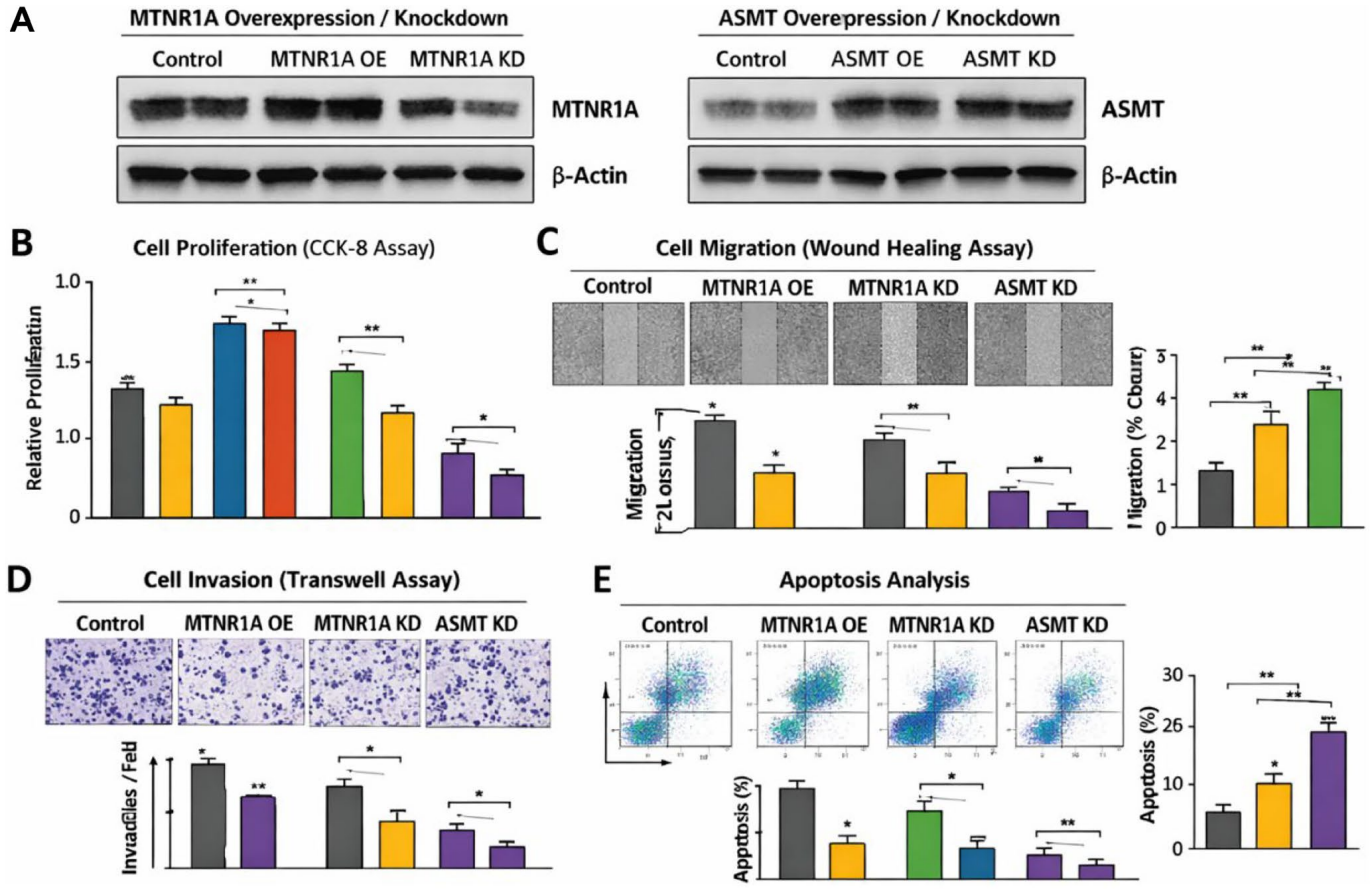
Gene Overexpression and Knockdown of *MTNR1A* and *ASMT* Regulate Proliferation, Migration, Invasion and Apoptosis in Glioma Cells. Panel A: Immunoblot analysis of MTNR1A and ASMT protein expression in glioma cells after gene overexpression (OE) or knockdown (KD). *β*‐actin was used as the loading control. Panel B: Cell Proliferation Assay (CCK‐8) results showing the relative proliferation of glioma cells with *MTNR1A* overexpression (MTNR1A OE), *MTNR1A* knockdown (MTNR1A KD), *ASMT* overexpression (ASMT OE) and ASMT knockdown (ASMT KD) compared to control cells. Statistical differences are indicated by asterisks. Panel C: Cell Migration Assay (Wound Healing Assay) demonstrating the migration ability of glioma cells. Representative images of cell migration (left) and quantification of migration (right) in MTNR1A and ASMT overexpressed and knocked down glioma cells. Statistical significance is shown with asterisks. Panel D: Cell Invasion Assay (Transwell Assay) showing the invasive capacity of glioma cells. Representative images of invading cells (left) and quantitative analysis of the number of cells that invaded the membrane (right) in control, MTNR1A OE, MTNR1A KD, ASMT OE and ASMT KD groups. Panel E: Flow cytometry–based apoptosis assay showing representative plots and quantification of apoptotic cells after MTNR1A and ASMT manipulation. Statistical comparisons among experimental groups were performed using one‐way ANOVA followed by Tukey's post hoc test. Data are presented as mean ± SD. *p < 0.05, **p < 0.01.

Functional assays revealed that restoration of MTNR1A or ASMT expression significantly altered cellular behaviours linked to melatonin signalling. Overexpression of *MTNR1A* or *ASMT* suppressed glioma cell proliferation, migration and invasion, while promoting apoptotic responses. Conversely, knockdown of *MTNR1A* or *ASMT* enhanced proliferative capacity, migratory activity, and invasive potential, accompanied by reduced apoptosis. These effects were consistently observed across multiple assays, including CCK‐8 proliferation analysis, wound‐healing migration assays, Transwell invasion assays, and flow cytometry–based apoptosis measurements. The functional consequences of MTNR1A and ASMT overexpression or knockdown are summarized in Figure 5.

**FIGURE 6:**
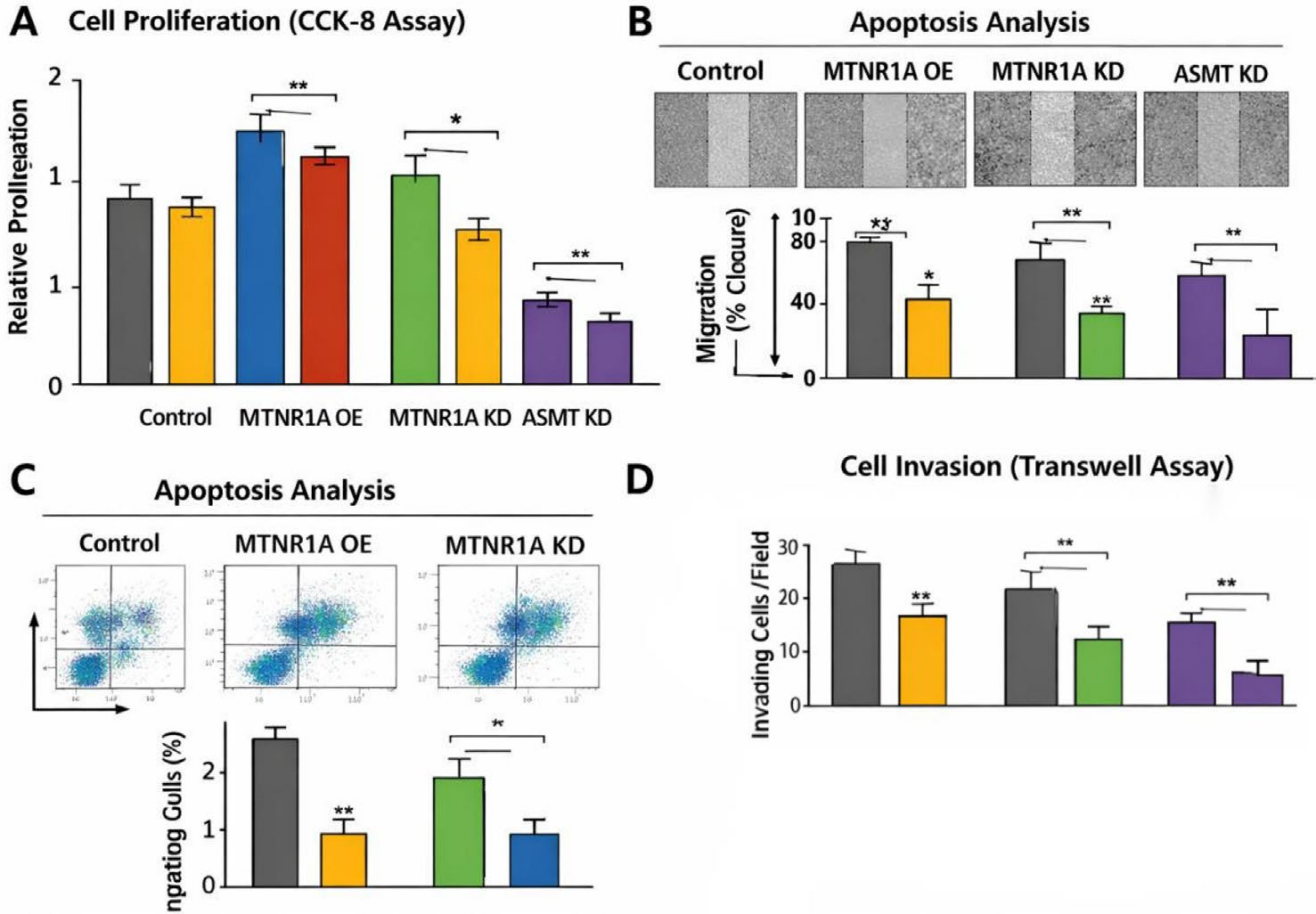
Effects of MTNR1A and ASMT modulation on glioma cell behaviour. Panel A: Cell proliferation was assessed using the CCK‐8 assay in glioma cells under different genetic modifications, including control, *MTNR1A* overexpression (OE), *MTNR1A* knockdown (KD) and *ASMT* knockdown (KD). Panel B: Representative wound‐healing assay images and quantitative analysis of migration ability, measured as the percentage of wound closure, are shown for each condition. Panel C: Apoptosis was assessed by flow cytometry. Representative dot plots and quantitative analysis of apoptotic cell percentages are shown for each condition. Panel D: Cell invasion was assessed using the Transwell assay. Quantitative analysis of invaded cell numbers per field is shown for each condition. Statistical comparisons among experimental groups were performed using one‐way ANOVA followed by Tukey's post hoc test. Data are presented as mean ± SD. *p < 0.05, **p < 0.01.

Importantly, these phenotypic changes reflect functional consequences of impaired melatonin signalling rather than nonspecific cytotoxic effects, supporting a causal role for *MTNR1A‐* and *ASMT‐*dependent pathways in regulating cellular processes relevant to melatonin biology within the glioma context. A focused analysis highlighting MTNR1A‐centered phenotypic alterations is shown in Figure 6.

### 3.4 Loss of *MTNR1A* and *ASMT* Derepresses AKT, ERK, and STAT3 Signalling Pathways

**FIGURE 7:**
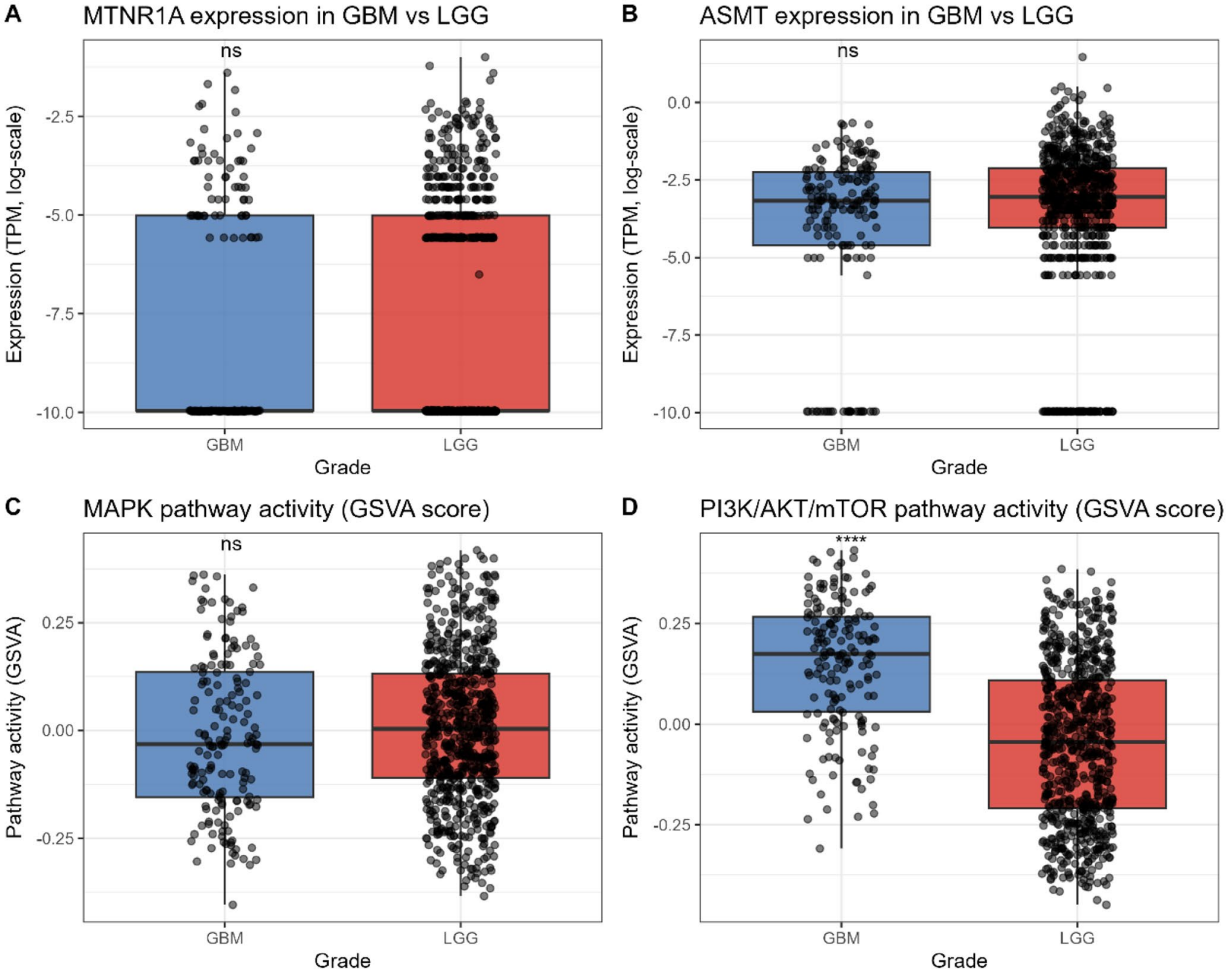
Association between *MTNR1A/ASMT* expression levels and pathway activity in glioma. Boxplots show the distribution of *MTNR1A* and *ASMT* expression and corresponding MAPK and PI3K/mTOR pathway activity scores in glioblastoma (GBM) and lower‐grade glioma (LGG). Samples were stratified into low‐ and high‐expression groups based on the first and third quartiles (Q1 and Q4). Boxes represent the interquartile range with median lines, and whiskers indicate data dispersion. Statistical comparisons between low‐ and high‐expression groups were performed using unpaired two‐tailed Student's *t*‐test or the two‐sided Wilcoxon rank‐sum test, as appropriate. *p* < 0.05 was considered statistically significant.

**FIGURE 8:**
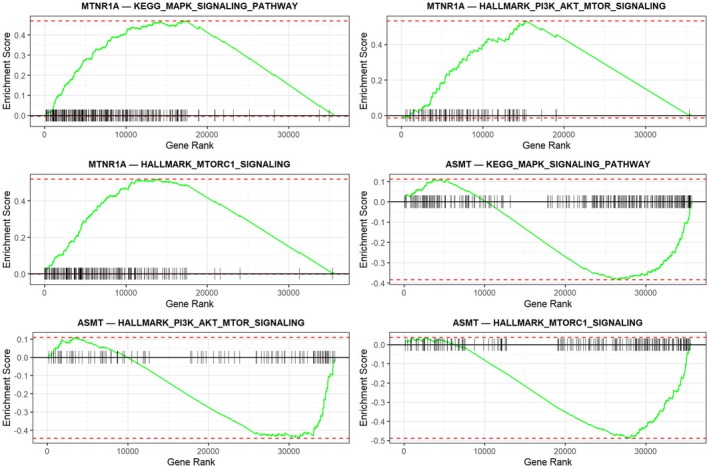
Gene set enrichment analysis of melatonin‐axis gene expression in glioma. Representative gene set enrichment analysis (GSEA) plots illustrating pathways enriched in glioma samples stratified by high versus low expression of *MTNR1A, AANAT*, and *ASMT*. Enrichment scores and normalized enrichment scores (NES) are shown for significantly associated pathways, including MAPK signalling, PI3K/AKT/mTOR signalling, and hypoxia‐related gene sets. GSEA was performed using permutation‐based testing, and pathways with a false discovery rate (FDR) < 0.05 were considered statistically significant.

Associations between *MTNR1A* and *ASMT* expression levels and downstream pathway activity across glioma grades are shown in Figure 7. Given the established role of melatonin receptors in regulating intracellular signalling cascades, we next examined whether disruption of MTNR1A and ASMT influenced downstream oncogenic signalling pathways predicted by transcriptomic enrichment analyses. Gene set variation analysis (GSVA) and gene set enrichment analysis (GSEA) of patient‐derived datasets indicated activation of PI3K/AKT, ERK/MAPK, and STAT3‐related pathways in gliomas with low *MTNR1A* and *ASMT* expression. Representative gene set enrichment analysis plots are shown in Figure 8.

**FIGURE 9:**
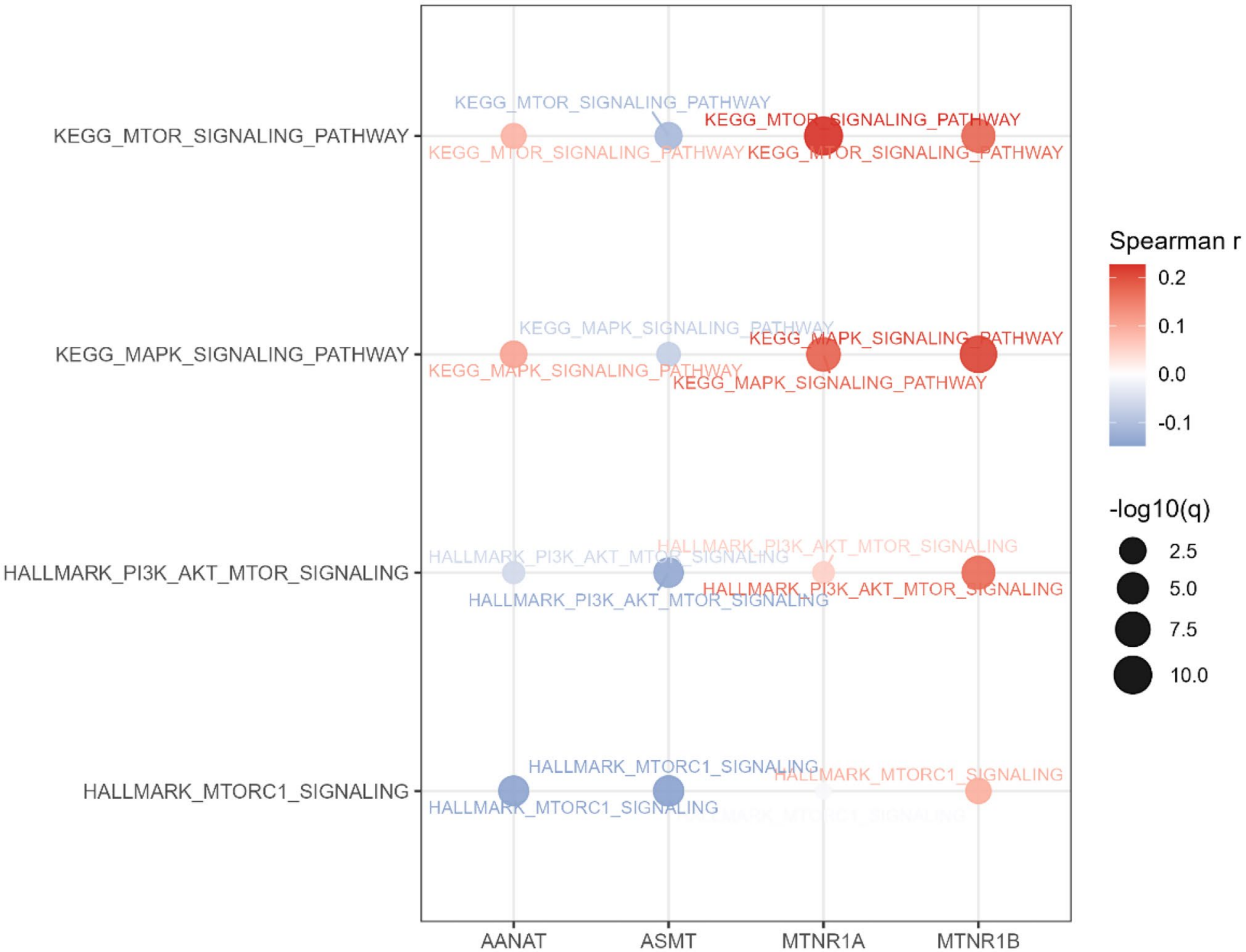
Correlation between melatonin‐axis gene expression and pathway activity in glioma. Bubble plot showing Spearman correlations between melatonin‐axis gene expression (*MTNR1A*, *MTNR1B*, *AANAT*, *ASMT*) and pathway activity scores derived from gene set variation analysis (GSVA). Correlations were assessed using Spearman correlation analysis. *p* values were adjusted using the Benjamini–Hochberg false discovery rate method. Each bubble represents a pathway, with colour indicating correlation strength, Spearman's r, and size reflecting statistical significance −log10 adjusted q value. Pathways meeting the significance thresholds |r| ≥ 0.2 and adjusted q < 0.05 are labelled.

**FIGURE 10:**
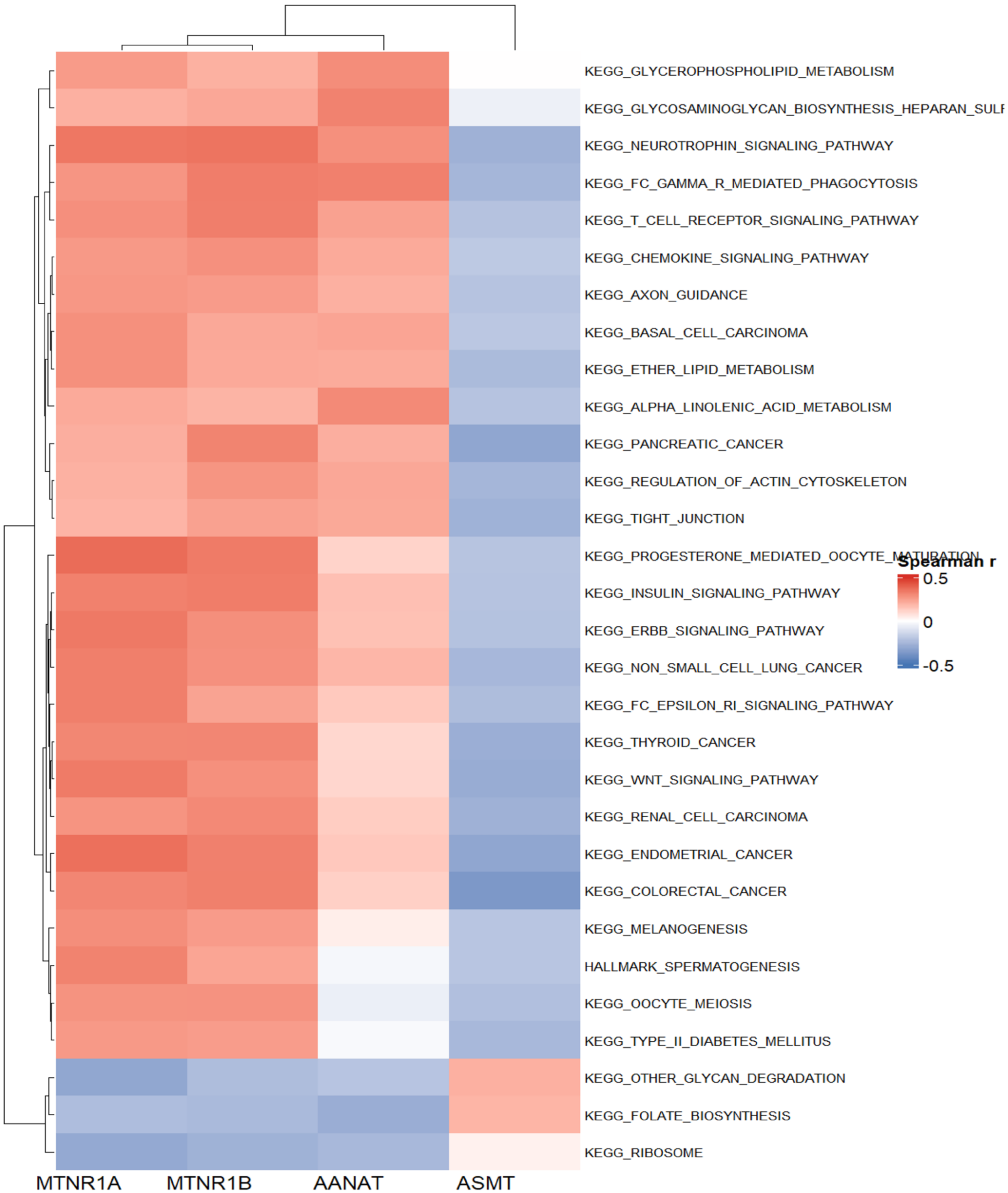
Heatmap of correlations between melatonin‐axis gene expression and downstream pathway activity. Heatmap depicting Spearman correlation coefficients between melatonin‐axis gene expression and GSVA‐derived pathway activity scores across glioma samples. Rows represent KEGG and Hallmark pathways, and columns correspond to pathway activity profiles across samples. Colour gradients indicate correlation direction and magnitude. Pathways with significant associations (|r| ≥ 0.2, adjusted q < 0.05) are highlighted. Correlation analysis was performed using Spearman's correlation coefficient, and *p* values were adjusted using the Benjamini–Hochberg false discovery rate method.

Correlation patterns between melatonin‐axis gene expression and pathway activity scores across glioma samples are summarized in Figure 9, and an integrated heatmap depicting pathway‐level associations is shown in Figure 10.

**FIGURE 11:**
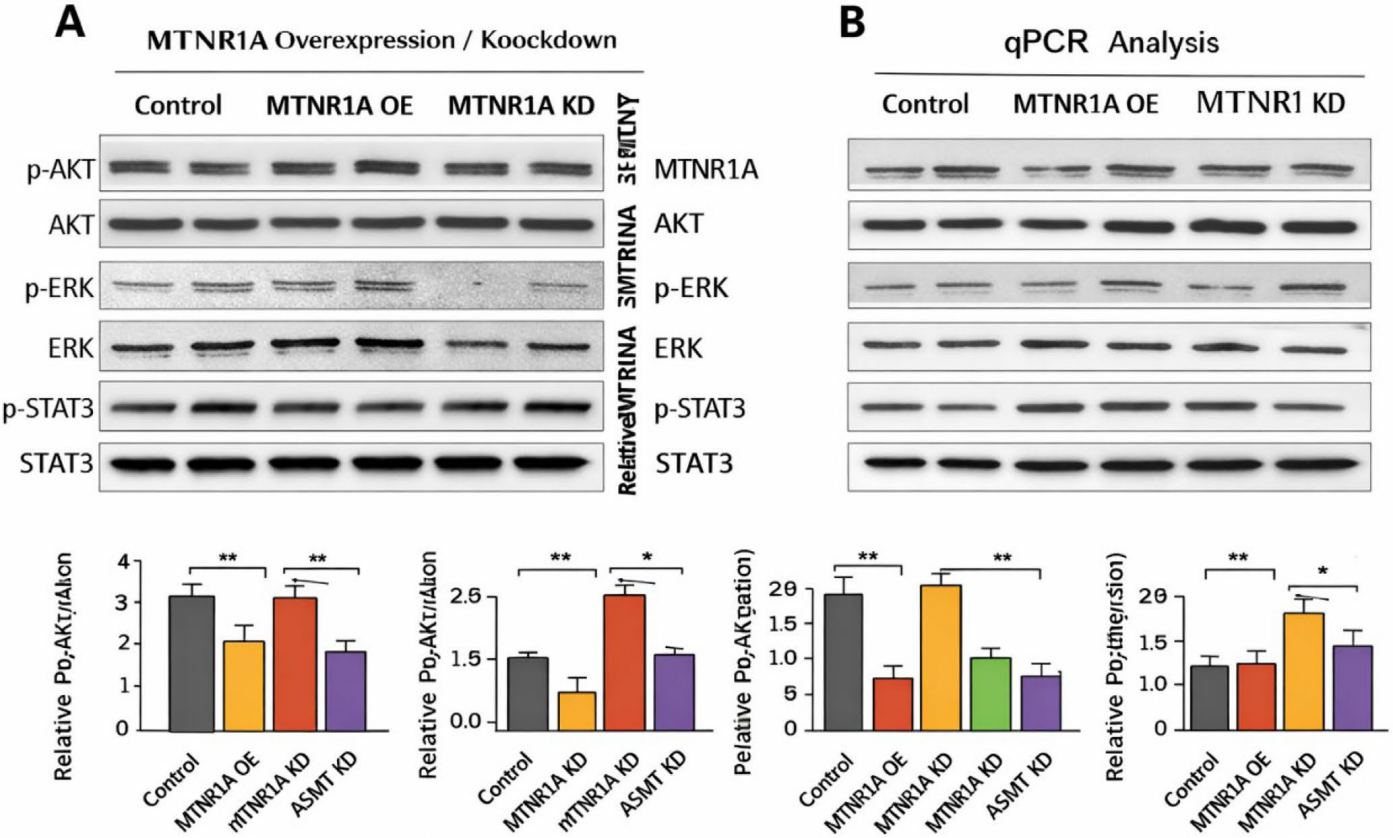
Signal Pathway Validation of *MTNR1A* and *ASMT* Modulation. Panel A: Western Blotting. Western blot analysis of key signalling proteins, including p‐AKT, AKT, p‐ERK, ERK, p‐STAT3 and STAT3, in glioma cells with *MTNR1A* overexpression (OE), *MTNR1A* knockdown (KD), and *ASMT* knockdown (KD). Protein levels were quantified and normalised against *β*‐actin. Panel B: QPCR Analysis. Quantitative PCR analysis of mRNA expression levels of *AKT, ERK* and *STAT3* in glioma cells with *MTNR1A* overexpression (OE), *MTNR1A* knockdown (KD), and *ASMT* knockdown (KD). Data were normalized to GAPDH. Statistical comparisons among experimental groups were performed using one‐way ANOVA followed by Tukey's post hoc test. Data are presented as mean ± SD. Significant differences are indicated by asterisks: *p < 0.05, **p < 0.01. p‐AKT, phosphorylated AKT; p‐ERK, phosphorylated ERK; p‐STAT3, phosphorylated STAT3.

Consistent with these in silico predictions, experimental modulation of *MTNR1A* and *ASMT* resulted in corresponding changes in key signalling nodes. Overexpression of *MTNR1A* led to reduced phosphorylation of AKT, ERK and STAT3, whereas knockdown of *MTNR1A* or *ASMT* increased phosphorylation of these signalling molecules. Total protein levels of AKT, ERK and STAT3 remained largely unchanged, indicating that melatonin‐axis disruption primarily affects pathway activation rather than protein abundance. Quantitative PCR analyses further supported these findings, demonstrating concordant changes in pathway‐associated gene expression. Experimental validation of AKT, ERK and STAT3 pathway modulation is shown in Figure 11.

Collectively, these results demonstrate that loss of receptor‐ and synthesis‐dependent melatonin signalling components derepresses AKT–ERK–STAT3 signalling, providing a mechanistic link between melatonin‐axis remodelling and downstream signalling alterations observed in human glioma.

### 3.5 Integrated Model of Melatonin‐Axis Disruption and Signalling Consequences in Glioma

Integrating transcriptomic profiling, protein‐level validation, functional assays, and signalling analyses, we propose a model in which hierarchical disruption of the tryptophan–melatonin axis compromises melatonin signalling integrity in glioma. Preferential loss of MTNR1A and ASMT reduces both receptor‐mediated responsiveness and endogenous melatonin synthesis, leading to derepression of AKT, ERK and STAT3 signalling pathways. This integrated framework positions human glioma as a disease context that reveals fundamental principles of melatonin‐axis regulation and its downstream signalling consequences.

## 4 Discussion

In this study, we investigated the integrity of the tryptophan–melatonin signalling axis in the human brain using glioma as a pathological context. By integrating large‐scale transcriptomic analyses with protein‐level validation, cell‐based functional assays and downstream signalling interrogation, we demonstrate that melatonin signalling is hierarchically disrupted in glioma. This disruption is characterized not by uniform suppression of all melatonin‐related components, but by preferential loss of receptor‐dependent signalling capacity, particularly *MTNR1A*, together with depletion of the synthesis‐limiting enzyme ASMT. These findings provide a biologically coherent framework for understanding how melatonin signalling integrity is compromised in human brain disease.

### 4.1 Hierarchical Disruption of Receptor‐ and Synthesis‐Dependent Melatonin Signalling

Melatonin signalling is organized along two interdependent layers: receptor‐mediated signal transduction and endogenous melatonin biosynthesis [1, 3]. Our data reveal that these layers are differentially affected in glioma. *MTNR1A* expression is markedly reduced with increasing tumour grade, while *MTNR1B* expression remains uniformly low across tissues, suggesting asymmetric vulnerability of melatonin receptor subtypes [4, 5, 19]. In parallel, ASMT‐widely regarded as a rate‐limiting enzyme in melatonin synthesis‐is consistently downregulated, whereas *AANAT* expression is relatively preserved, particularly in lower‐grade glioma. This pattern indicates that loss of melatonin signalling integrity arises from selective disruption of critical nodes rather than complete pathway collapse.

Such hierarchical remodelling has important biological implications. Loss of MTNR1A receptor expression diminishes cellular responsiveness to melatonin, even in the presence of circulating or locally synthesized hormone, whereas ASMT depletion likely restricts endogenous melatonin availability [1, 9, 20]. Together, these alterations suggest that melatonin deficiency in glioma reflects impaired signalling competence rather than absolute absence of melatonin, a distinction that is often overlooked but central to melatonin biology.

### 4.2 Functional Consequences of Melatonin‐Axis Disruption

Beyond descriptive profiling, our functional experiments provide causal evidence that MTNR1A and ASMT are biologically active regulators of cellular behaviour associated with melatonin signalling [21, 22, 23]. Experimental restoration of *MTNR1A* or *ASMT* attenuated proliferation, migration and invasion while promoting apoptosis, whereas their depletion exerted opposing effects. Importantly, these phenotypes reflect alterations in melatonin‐dependent cellular regulation rather than nonspecific cytotoxicity, supporting a direct functional role of these components [24, 25, 26, 27].

These observations align with prior experimental studies demonstrating that melatonin and its receptors influence cellular homeostasis, oxidative stress responses, and survival pathways in neural and cancer cells. Our findings extend this literature by demonstrating that disruption of melatonin‐axis components in human disease is sufficient to reprogram cellular behaviour, thereby bridging the gap between transcriptomic associations and biological function.

### 4.3 Derepression of AKT–ERK–STAT3 Signalling Links Melatonin‐Axis Loss to Downstream Pathways

Melatonin receptors are known to modulate multiple intracellular signalling cascades, including PI3K/AKT and MAPK/ERK pathways [4, 8, 28]. Consistent with this biology, pathway enrichment analyses revealed activation of AKT‐, ERK‐ and STAT3‐related signalling in gliomas with low *MTNR1A* and *ASMT* expression [20, 29, 30]. Experimental validation confirmed that loss of these melatonin‐axis components leads to increased phosphorylation of AKT, ERK, and STAT3, while restoration suppresses pathway activation.

These findings support a mechanistic model in which melatonin‐axis disruption removes inhibitory constraints on pro‐survival and stress‐response signalling pathways. Importantly, changes were observed primarily at the level of phosphorylation rather than total protein abundance, indicating that melatonin signalling modulates pathway activity rather than gene expression alone [6, 31, 32, 33]. This phosphorylation‐dominant regulatory pattern may involve both receptor‐dependent and metabolism‐dependent mechanisms. As MTNR1A is a classical G protein–coupled melatonin receptor, reduced MTNR1A receptor expression may directly impair melatonin‐mediated receptor signalling and thereby alter downstream kinase activity, including AKT, ERK and STAT3 phosphorylation. In contrast, ASMT is a key enzyme involved in melatonin biosynthesis; therefore, ASMT deficiency may reduce endogenous melatonin production and indirectly reshape tryptophan metabolic flux. Such metabolic remodelling may weaken melatonin‐dependent inhibitory signalling while potentially favouring alternative tryptophan catabolic pathways, thereby further facilitating oncogenic kinase activation. Thus, the increased phosphorylation of AKT, ERK and STAT3 observed after *MTNR1A* or *ASMT* suppression may reflect a combination of direct receptor‐coupled signalling loss and indirect metabolic shifts within the tryptophan–melatonin pathway. However, because receptor‐specific antagonists, melatonin rescue experiments, and metabolomic profiling were not performed in the present study, the relative contribution of these two mechanisms requires further investigation. This signalling derepression provides a plausible molecular link between melatonin‐axis remodelling and broader cellular and metabolic reprogramming observed in glioma.

### 4.4 Implications for Tryptophan Metabolism and Immune–Metabolic Balance

Tryptophan metabolism represents a critical metabolic branching point in the brain, balancing serotonin–melatonin synthesis against diversion toward the kynurenine pathway [1, 5, 34]. Disruption of melatonin synthesis through ASMT loss may shift this balance, favouring kynurenine accumulation and downstream activation of aryl hydrocarbon receptor–dependent immunoregulatory pathways. In glioblastoma, increased kynurenine pathway activity may contribute to an immunosuppressive tumour microenvironment by promoting immune‐tolerant phenotypes, suppressing anti‐tumour T‐cell responses, and facilitating tumour‐associated macrophage or microglial polarization. This metabolic shift may therefore provide an additional mechanism by which loss of the tryptophan–melatonin axis supports glioma progression beyond direct activation of AKT, ERK, and STAT3 signalling. Although not directly assessed in this study, this possibility offers a broader framework linking melatonin‐axis disruption to immune–metabolic dysregulation in glioblastoma. Within this context, glioma serves as a human disease model revealing how perturbations of the tryptophan–melatonin axis may contribute to altered neuroimmune signalling [25, 35, 36]. Future studies integrating metabolomic profiling and immune analyses will be essential to fully elucidate these interactions.

### 4.5 Circadian and Translational Considerations

Melatonin signalling is intrinsically linked to circadian regulation [12, 37]. While our study did not explicitly account for time‐of‐day effects or circadian phase, the observed loss of melatonin signalling integrity has potential implications for chrono‐therapeutic strategies [5, 30]. Importantly, chronotherapeutic interventions rely not only on the external timing of treatment but also on the preserved ability of tumour cells to respond to rhythmic circadian cues. The static loss of receptor integrity, particularly reduced MTNR1A receptor expression, may blunt the dynamic responsiveness of glioma cells to endogenous nocturnal melatonin peaks or timed exogenous melatonin administration. In this context, even appropriately timed melatonin‐based interventions may be less effective in tumours with profound receptor loss. Conversely, tumours retaining MTNR1A receptor expression but showing impaired biosynthetic capacity may remain more responsive to exogenous melatonin or chronotherapy‐based strategies. Thus, stratification based on melatonin‐axis integrity may help identify patients more likely to benefit from melatonin‐based interventions and may inform the optimal timing of chronotherapeutic strategies [16, 17, 31].

### 4.6 Limitations

Several limitations warrant consideration. First, circadian timing and dynamic oscillations of melatonin signalling were not evaluated. Second, in vivo validation was beyond the scope of this study. Nevertheless, the convergence of transcriptomic, protein‐level, functional and signalling data provides robust evidence supporting hierarchical disruption of the melatonin axis in human glioma. These findings establish a foundation for future mechanistic and translational investigations.

## 5 Conclusions

In summary, our study demonstrates that melatonin signalling integrity is hierarchically compromised in glioma, characterized by preferential loss of MTNR1A receptor‐mediated signalling and ASMT‐dependent melatonin synthesis. Using glioma as a human disease model, we reveal how disruption of the tryptophan–melatonin axis leads to derepression of key intracellular signalling pathways, with broad implications for circadian biology, neuroendocrine regulation and melatonin‐based therapeutic strategies. These findings advance understanding of melatonin‐axis biology in the human brain and highlight the importance of preserving signalling integrity rather than hormone availability alone.

## Author Contributions

**Xiaohong Yin:** formal analysis, funding acquisition, project administration. **Peng Song:** writing – original draft. **Suqiu Yao:** conceptualization, data curation, methodology, resources. **Shuangyin He:** data curation, methodology, resources. **Bo Tan:** conceptualization, data curation, formal analysis, visualization, writing – original draft, writing – review and editing, project administration, supervision, investigation, methodology, software, validation, funding acquisition, resources. **Ying Chen:** writing – review and editing, methodology, software, data curation. **Han Chen:** data curation, resources. **Tao Chen:** validation, resources, methodology. **Wenfu Yang:** data curation, resources. **Tingting Xu:** software, funding acquisition. **Jiajie Zhang:** data curation, resources, project administration. **Xiyuan Tang:** data curation, resources.

## Funding

This research was supported by the following: Health Commission of Sichuan Province Medical Science and Technology Program (Grant No. 24WSXT042). Wu Jieping Medical Foundation (Grant No. 320.6750.2024‐6‐113). Sichuan Provincial Clinical Key Specialty Construction Project (Grant No. 2024HSWKP001). Guangyuan Science and Technology Bureau—Science and Technology Project (Grant No. 23ZDYF0053).

## Ethics Statement

All analyses involving human transcriptomic data were conducted using publicly available, de‐identified datasets obtained from The Cancer Genome Atlas (TCGA), the Chinese Glioma Genome Atlas (CGGA), and the Genotype‐Tissue Expression (GTEx) project. Archived human tissue specimens were obtained from Guangyuan Central Hospital and were anonymized prior to analysis. According to institutional policies, retrospective studies using anonymized archived specimens that do not involve direct patient contact or intervention are exempt from formal ethical approval. No new human participants or animals were recruited for this study.

## Conflicts of Interest

The authors declare no conflicts of interest.

## Data Availability

The data that support the findings of this study are available from the corresponding author upon reasonable request.

## References

[1] R. J. Reiter , R. Sharma , S. Rosales‐Corral , et al., “Melatonin: Biology, Physiology and Pharmacology in Health and Disease,” Pharmacology & Therapeutics 224 (2022): 107837, 10.1016/j.pharmthera.2021.107837.

[2] R. J. Reiter , D. X. Tan , A. Korkmaz , et al., “Melatonin as an Antioxidant: Molecular Mechanisms and Clinical Significance,” Acta Biochimica Polonica 82 (2022): 101743, 10.1016/j.arr.2022.101743.

[3] S. Comai and G. Gobbi , “Melatonin, Melatonin Receptors and Sleep: Moving Beyond Traditional Views,” Journal of Pineal Research 76, no. 7 (2024): e13011, 10.1111/jpi.13011. 39400423

[4] G. S. Kinker , S. M. Oba‐Shinjo , P. O. Carvalho , et al., “MT1 and MT2 Melatonin Receptors Play Opposite Roles in Human Glioma/Medulloblastoma Cells,” Journal of Pineal Research 70, no. 2 (2021): e12718, 10.1111/jpi.12718. 33503294

[5] G. S. Kinker , K. G. Kinker , L. G. de Almeida Chuffa , et al., “Melatonin Receptors MT1 and MT2 Exert Distinct Effects on Glioma Cell Proliferation and Migration Through Modulation of ERK and AKT Signaling,” Journal of Pineal Research 70, no. 2 (2021): e12703, 10.1111/jpi.12703. 33125735 PMC7816253

[6] A. Davoodvandi , M. Biglar , A. Allahverdi , et al., “Melatonin and Cancer Suppression: Insights Into Molecular Mechanisms,” Cancers (Basel) 14, no. 15 (2022): 3778, 10.3390/cancers14153778. 35954440 PMC9367439

[7] J. Yan , H. Zhang , Z. Wang , et al., “Tryptophan Metabolism in Cancer: Therapeutic Significance,” Signal Transduction and Targeted Therapy 9, no. 1 (2024): 190, 10.1038/s41392-024-01755-3.39039046

[8] G. Anderson and R. J. Reiter , “Melatonin, BAG‐1 and Cortisol Circadian Interactions in Tumor Pathogenesis,” Biomedicine & Pharmacotherapy 165 (2023): 115035, 10.1016/j.biopha.2023.115035. 37970210 PMC10645470

[9] M. Ghareghani , K. Zibara , R. J. Reiter , and S. Rivest , “Reduced Melatonin Levels May Facilitate Glioblastoma Initiation in the SVZ,” Expert Reviews in Molecular Medicine 24 (2022): e24, 10.1017/erm.2022.15. 35570582

[10] Y. Liu , Y. Xiang , Q. Liao , L. Peng , and J. Shen , “Tryptophan Metabolic Gene‐Related Risk Signature in Gliomas,” Frontiers in Oncology 12 (2022): 1037705, 10.3389/fonc.2022.1037705.

[11] Q. T. Ostrom , G. Cioff , K. Waite , et al., “CBTRUS Statistical Report: Primary Brain and Other CNS Tumors Diagnosed in the United States in 2016–2020,” Neuro‐Oncology 25 (2023): iv1–iv99, 10.1093/neuonc/noad183. 37793125 PMC10550277

[12] J. M. Melhem , L. Gay , L. Jin , et al., “Updates in IDH‐Wildtype Glioblastoma,” Current Oncology Reports 24, no. 9 (2022): 1151–1165, 10.1007/s11912-022-01273-0.

[13] P. P. Segura , B. Ramos , S. Gil‐Robles , et al., “SEOM–GEINO Clinical Guidelines for High‐Grade Gliomas (2023),” Clinical & Translational Oncology 25, no. 10 (2023): 2511–2536, 10.1007/s12094-023-03208-4. PMC10425506 37540408

[14] F. Winkler , D. C. Hoffmann , V. Venkataramani , et al., “Autonomous Rhythmic Activity in Glioma Networks Drives Brain Tumor Growth,” Nature 613 (2023): 179–186, 10.1038/s41586-022-05520-4. 36517594

[15] M. F. González‐Aponte , B. Fekry , M. R. Salisbury , et al., “Circadian Regulation of MGMT Creates a Chronochemotherapy Window in GBM,” Science Advances 10, no. 20 (2024): eadk9220, 10.1126/sciadv.adk9220.

[16] M. Petković and B. Pejin , “Chronotherapy in Glioblastoma: State of the Art and Future Challenges,” Cancers (Basel) 15, no. 3 (2023): 807, 10.3390/cancers15030807. 36765764 PMC9913560

[17] Z. Wang , X. Zhang , X. Zhao , et al., “Circadian Clock in Glioma: Mechanisms to Chronotherapy,” Medicinal Research Reviews 42, no. 6 (2022): 2531–2571, 10.1002/med.21959.

[18] N. Nelson , “An Emerging Role for the Circadian Clock in Glioblastoma,” Npj Precision Oncology 8 (2024): 60, 10.1038/s41698-024-00530-z. 38378853 PMC10879494

[19] B. I. Fernández‐Gil , A. Rodríguez‐Rodríguez , A. González‐González , et al., “Melatonin Triggers Metabolic and Intracellular pH Imbalance in Glioblastoma,” Cells 11, no. 21 (2022): 3467, 10.3390/cells11213467. 36359862 PMC9654239

[20] F. Wang , Y. Zhu , S. Wanggou , et al., “Melatonin Enhances Nimotuzumab Efficacy by Inhibiting EGFR in Glioblastoma,” Cancer Letters 592 (2024): 216920, 10.1016/j.canlet.2024.216920. 38679408

[21] A. Bostanci and O. Doganlar , “Melatonin Enhances Temozolomide‐Induced Apoptosis in Glioblastoma and Neuroblastoma Cells,” Experimental Oncology 46, no. 2 (2024): 87–100, 10.15407/exp-oncology.2024.02.087. 39396175

[22] L. Chen , S. Li , X. Wang , et al., “Melatonin Synergises the Chemotherapeutic Effect of Temozolomide in Glioblastoma,” Journal of Neuro‐Oncology 168, no. 3 (2024): 455–468, 10.1007/s11060-024-04456-1.

[23] L. Olmedo‐Moreno , C. Panadero‐Morón , I. Fernández‐García , et al., “Melatonin‐Primed Mesenchymal Stromal Cells Hinder Glioblastoma Progression,” Theranostics 15, no. 6 (2025): 3076–3095, 10.7150/thno.92287. 40083939 PMC11898303

[24] Z. Yan , X. Zhang , L. Hua , and L. Huang , “Melatonin Inhibits Malignant Progression of Glioblastoma via miR‐16‐5p/PIM1,” Current Neurovascular Research 19, no. 1 (2022): 92–99, 10.2174/1567202619666220406084947. 35388757

[25] R. X. Wang , H. Liu , L. Xu , H. Zhang , and R. X. Zhou , “Melatonin Suppresses Glioblastoma via ER Stress Pathway,” Neurochemical Research 45, no. 9 (2020): 2081–2090, 10.1007/s11064-020-03049-7.

[26] J. Wang , C. Li , H. Zhao , et al., “Melatonin Inhibits Glioma Cell Growth and Sensitizes Cells to Temozolomide via Modulation of Endoplasmic Reticulum Stress and EGFR Signaling Pathways,” Neurochemical Research 45, no. 7 (2020): 1601–1612, 10.1007/s11064-020-03035-5.

[27] Y. Chen , X. Zhang , Z. Liu , et al., “Functional Role of Melatonin in Glioma Progression: Inhibition of Proliferation and Migration via EGFR/AKT/ERK Pathway Modulation,” Journal of Neuro‐Oncology 169, no. 1 (2024): 55–68, 10.1007/s11060-024-04352-2.

[28] J. M. Murray , S. W. Cain , E. M. McGlashan , et al., “At‐Home Salivary DLMO Protocol,” Journal of Pineal Research 76, no. 5 (2024): e12994, 10.1111/jpi.12994. 39158010

[29] E. A. Nettnin , L. E. Coate , and K. L. Pitter , “Therapeutic Approaches for Circadian Modulation in High‐Grade Glioma,” Neuro‐Oncology Advances 5, no. 1 (2023): iii3–iii12, 10.1093/noajnl/vdad127.

[30] K. Zappe , I. de Kock , M. Popovic , et al., “MGMT Enhancer Methylation Associates With MGMT Expression in Glioblastoma,” Cancers (Basel) 15, no. 13 (2023): 3395, 10.3390/cancers15133395. 37444505 PMC10340418

[31] L. Wang , L. Chen , Z. Zhou , et al., “Emerging Role of the Circadian Clock in Glioblastoma (Review),” npj Precision Oncology 8 (2024): 60, 10.1038/s41698-024-00530-z. 38378853 PMC10879494

[32] R. Perelroizen , B. Philosof , N. Budick‐Harmelin , et al., “Astrocyte Immunometabolic Regulation Governs Glioblastoma Progression,” Nature Cancer 3, no. 9 (2022): 1104–1122, 10.1093/brain/awac222. PMC10233310 35899587

[33] Y. Zhong , Z. Wang , J. Zhang , et al., “Combinatorial Targeting of Glutamine and Lysosomal Lipid Metabolism Suppresses Glioblastoma,” Signal Transduction and Targeted Therapy 9, no. 1 (2024): 244, 10.1038/s41392-024-01807-8.39278900

[34] X. Chen , J. Sun , Y. Li , et al., “Proteomic/Metabolomic Mechanisms Regulating MGMT in Glioblastoma,” CNS Neuroscience & Therapeutics 30, no. 2 (2024): e14415, 10.1111/cns.14415. 37641495 PMC10848106

[35] X. Li , H. Yu , L. Wang , et al., “Prognostic Value of Melatonin Receptors in Glioma: Meta‐Analysis,” BMC Cancer 21, no. 1 (2021): 679, 10.1186/s12885-021-08476-3.34107921

[36] Q. Zeng , X. Li , J. Chen , et al., “Single‐Cell RNA‐Seq Atlas of Glioblastoma Reveals Tumor–Microenvironment Interactions,” iScience 26, no. 5 (2023): 106781, 10.1016/j.isci.2023.106781. 37213226 PMC10199267

[37] K. Yao , X. Ma , W. Li , et al., “Spatial Transcriptomics Delineates Cellular Niches in Glioblastoma,” Cell Reports Medicine 5, no. 4 (2024): 101468, 10.1016/j.xcrm.2024.101468. 38508144 PMC10983111

